# Performance of p16/Ki67 Immunostaining for Triage of Elderly Women with Atypical Squamous Cells of Undetermined Significance

**DOI:** 10.3390/jcm12103400

**Published:** 2023-05-11

**Authors:** Maria Teresa Bruno, Arianna Guaita, Sara Boemi, Gabriele Mazza, Maria Chiara Sudano, Marco Palumbo

**Affiliations:** 1 Department of General Surgery and Medical Surgery Specialties, Gynecological Clinic, University of Catania, 95100 Catania, Italy; 2 Multidisciplinary Research Center in Papillomavirus Pathology, University of Catania, 95100 Catania, Italy; 3 Department of Statistics, Sapienza University of Roma, 00185 Rome, Italy

**Keywords:** ASC-US, HPV genotypes, p16/Ki67, CIN2+

## Abstract

Background: The p16/Ki67 technique has been poorly studied in postmenopausal women with ASC-US cytology. The objective of this study was to compare the accuracy of p16/Ki67 staining, HPV testing and HPV 16 genotyping for the identification of CIN2 + lesions in postmenopausal women with ASC-US cytology. Method: A total of 324 postmenopausal women with positive ASC-US were included. The women underwent HPV test, colposcopy, and biopsy. The slides were discolored and then stained with the CINtec Plus Kit for p16/Ki67. The HPV test results were classified as HPV16 +, hrHPV+ (other hrHPV genotypes), or HPV negative. Results: The p16/Ki67 sensitivity for CIN2+ was 94.5%, the specificity 86.6%, PPV of 59% and NPV of 95.9%. The HPV test showed a sensitivity of 96.4% for CIN2+, a specificity of 62.8%, a PPV of 35% and a NPV of 98.8%. In postmenopausal women, the prevalence of genotype 16 decreases in favor of the other high-risk genotypes. Conclusion: Given the low sensitivity of cytology and the low percentage of HPV16-positive cancers among elderly women, triage via cytology and genotyping is not the best strategy; double staining cytology shows high profiles of sensibility and specificity for CIN2+ in ASCUS postmenopausal women.

## 1. Introduction

The age-related incidence of cervical carcinoma reported in Germany [1] in 2013 was the highest in the age groups 35–49 years and 50–64 years (16.5 per 100,000 and 14.8 per 100,000, respectively). Similar estimates have been reported in other populations in developed countries such as the United Kingdom and Australia [2,3]. Nearly one in four cases are diagnosed after age 60 and have a worse prognosis [4]. Older women are at greater risk of invasive cervical cancer due to the difficulty of early recognition and interpretation of dysplastic abnormalities that may appear. The cytological anomaly that raises the most concerns in both young and older women is certainly the Atypical Squamous Cells of Undetermined Significance (ASC-US). ASC-US is an ambiguous cytological result; it is not a true diagnostic entity. The term ASCUS includes a series of alterations of inflammatory, reactive, metaplastic and even preneoplastic origin with a risk of CIN2+ of 9.7% [5]. In postmenopausal women, the diagnosis of ASC-US is two times greater than that in women of childbearing age [6]. In recent years, there has been an increase in postmenopausal women sent to the second level for ASC-US cytology, increasing the amount of work of colposcopy centers, increasing health expenditure as well as anxiety in the patient and her family.

The management of ASC-US was a problem until the publication of the ASC-US/Low-grade Squamous Intraepithelial Lesion Triage Study (ALTS Study), in which the HPV test was incorporated into the interpretation and management process [7]. The American Society for Colposcopy and Cervical Pathology (ASCCP), based on the ALTS study, recommends an approach with HPV testing for those patients who have a diagnosis of ASC-US [8]. This principle also applies to postmenopausal ASC-US-positive women, because the Pap smear test and colposcopy, the most common diagnostic methods, have a limited use in menopause. Today the hrHPV Test, thanks to its high sensitivity, is the primary screening test for women over 35 years old, and cytology, with higher specificity than the HPV test, is used as triage for positive cases.

In the literature, the vast majority of ASC-US hrHPV-positive cases did not have underlying high-grade abnormalities [5].

Short-term estrogen treatment, followed by a repeat Pap smear test, can correct false positive interpretations of cytology [9]; however, it would be preferable to have a biomarker that provides a sensitivity similar to that of the HPV test, but with significantly higher specificity, thus reducing rates of referral to colposcopy. The p16 protein, a cyclin-dependent kinase inhibitor that regulates the cell cycle, is expressed at very low concentrations in normal cells, but is strongly overexpressed when RB is inactivated by E7 of HPV [10]. When p16 is overexpressed in cells, a brown cytoplasmic and/or nuclear staining is produced. Ki67, a nuclear protein and marker of proliferation, is detected only in dividing cells (G1, S, G2 and M phase) and not in quiescent cells (G0 phase) [11]. In cases of overexpression, Ki67 manifests itself as a red nuclear spot.

The co-expression of p16 and Ki67 within the same cell occurs only under abnormal conditions [12]. Many studies point to the overexpression of such proteins in the cell as an excellent marker for CIN2+, but little has been studied in postmenopausal women with ASC-US cytology [13].

The objective of this study was to compare the accuracy of p16/Ki67 staining in the HPV DNA test and in HPV 16 genotyping for the identification of CIN2+ lesions in postmenopausal women with ASC-US cytology.

## 2. Materials and Methods

### 2.1. The Study Population

Between 2017 and 2020, 856 women were diagnosed with ASC-US on cytology. From the database of our HPV Unit center, we enrolled 493 post-menopausal women (age: 54.9 ± 5.0 years) for at least two years, with a cytological diagnosis of ASCUS and subjected to HPV test triage. The results of the HPV test were classified into HPV16 positive, hrHPV positive (other hrHPV genotypes) or HPV negative. Patients with a history of cancer, those with chronic or autoimmune diseases, those who had received hormone replacement therapy, and those who had not completed the diagnostic pathway were excluded.

The p16/Ki67 test was performed retrospectively on all conventional pap smears diagnosed with ASC-US and where discoloration could be performed. All slides were reviewed by two gynecological pathologists.

The results were compared with the histological results of the patients’ biopsy or cone biopsy with LEEP. We performed 176 targeted biopsies in Transformation Zone 1–2 and 149 diagnostic LEEPs in Transformation Zone 3.

The histological diagnosis of CIN was established according to the criteria of the World Health Organization on the basis of morphological criteria using H&E staining.

The primary outcome was Sensitivity (SE), Specificity (SPE), NPV and PPV of p16/Ki67 for CIN2+ compared with those of the HPV test in post-menopausal ASC-US women. The secondary outcome was the significance of HPV16 genotyping in the triage of post-menopausal women with ASC-US.

The study protocol was notified, according to the current legislation on observational studies provided by AIFA, to the Catania1 Ethics Committee of the Catania University Hospital, which did not request additions or changes to the protocol. Furthermore, the Catania1 Ethics Committee of the Catania University Hospital found the consent of the study participants unnecessary as the study concerned only the retrospective review of the medical database

### 2.2. HPV Test and Genotyping

After cytological sampling for HPV DNA, samples were sent to the laboratory for DNA extraction and viral DNA genotyping via genetic amplification followed by hybridization with genotype-specific probes capable of identifying most of the HPV genotypes of the genital region [18 high-risk HPV genotypes (16, 18, 26, 31, 33, 35, 39, 45, 51, 52, 53, 56, 58, 59, 66, 68, 73, 82), 7 low risk (6, 11, 40, 43, 44, 54, 70) and 3 undefined risk (69,71,74)]. The commercial method used was the MAG NucliSenseasy system (bioMérieux SA, Marct l’Etoile, France). The technique was previously described [14].

### 2.3. Colposcopy

Colposcopy was performed using a Zeiss OPM1F colposcope (Carl Zeiss, Jena, Germany) and applying acetic acid and a Lugol iodine solution. Any colposcopic anomaly was classified according to the nomenclature proposed by the International Federation for Colposcopy and Cervical Pathologies (IFCPC) into three degrees of increasing abnormality according to severity: (i) Abnormal Transformation Zone (ATZ) grade 1 (ATZ1); (ii) grade 2 (ATZ2); or (iii) cancer. We evaluated the visibility of the squamous-columnar junction (SCJ) and specific biopsies were taken from the portion.

### 2.4. p16/Ki67 Immunocytochemistry

Double p16/Ki67 immunostaining was performed retrospectively on all Pap smears interpreted as ASC-US with the CINtec Plus Kit (Roche mtm laboratories AG, Heidelberg, Germany) according to the manufacturer’s instructions. One of the salient features of the test is that it does not require additional samples and can be used on the same slide set up for the Pap smear. We performed the discoloration of the Pap smears, and then, we colored the entire slide.

#### Cytological Slides: Decolorization Protocol

Disassemble the slide with xylene for about 12 h and perform four passages in alcohol of 5 min at decreasing concentrations (100%, 95%, 80%, 70%); then, perform the decolorization: 5 min in 70% alcoholic solution, keep in H_2_O for 5 min and begin the first passage in 70% alcohol (5–10 min), then in 80% alcohol and then in H_2_O.

A positive reaction was defined if a red-colored nucleus and brown-colored cytoplasm were present in the same cell, no matter how abnormal the cell morphology was.

Cells with cytoplasmic brown staining only and/or nuclear red staining only were defined as negative.

### 2.5. Statistical Analysis

The analyses were conducted with the STATA software version 13 (StataCorp version 13. College Station, TX, USA). The sample was analyzed using descriptive statistics, using the median, mean and standard deviation for quantitative variables, absolute frequencies and percentages for categorical ones. The results are summarized in figures and tables. The homogeneity of the groups was evaluated using the *t*-test for the difference between averages or proportions, the association between p16/Ki67 and the presence of CIN2+ using the Chi-square test and Fisher Exact test for tables with dimensions <5. Using a histological diagnosis of CIN2+ as an end-point, we examined the sensitivity and specificity with widths of 95% confidence intervals (95% CI) of the HPV Test and p16/Ki67 immunocytology in post-menopausal women with ASC-US. We also analyzed the significance of HPV16 genotyping in post-menopausal women. The value of *p* < 0.05 was considered as statistical significance for all analyses.

## 3. Results

Forty-two of the 493 enrolled women were excluded due to inadequate slides. In addition, the ASCUS slides, before double staining, were reevaluated with the following result: 49 cases of atrophy, 39 cases of reactive alterations and 3 cases of HSIL were excluded. Therefore, a total of 324 patients were considered (Figure S1).

Of the 324 postmenopausal women in the study, 153 (47.3%) were HPV positive and 171 (52.7%) were HPV negative (Table 1). Considering the HPV-positive women, 53 experienced CIN2+ lesions; the remaining 100 women had a significant proportion of low-grade or reactive (CIN1/negative) lesions. Among HPV-negative women, only two cases of CIN2+ lesions were observed (χ^2^ = 64.188; *p* < 0.001) OR = 44.79 (95% CI 10.68–187.77) (Table 1). In conclusion, we had 55 cases (16.9%) of CIN2+ lesions (35 cases of CIN2, 18 cases of CIN3 and 2 cases of squamous cell carcinoma).

Stratifying the 153 HPV-positive women according to genotyping, 74 (48.4%) cases were HPV16+ and 79 (51.6%) cases were other high-risk genotypes (hrHPV+) positive (χ^2^ = 2.482; *p* > 0.05) OR: 0.58 (95% CI 0.30–1.14) (Table 2).

Among women positive for genotype 16 only, 21 cases (28.4%) were positive for a CIN2+ lesion.

Among other hrHPV-positive women, we found 32 cases (40.5%) of CIN2+ lesions. The most frequent genotype was 31 (8 cases), followed by HPV33 (7 cases), HPV52 (5 cases), HPV51 (5 cases), HPV35 (4 cases), HPV45 (2 cases) and HPV18 (1 case).

The prevalence of genotype 16 in postmenopausal women with CIN2+ histological lesions was 38.2% (21/55), and that for hrHPV+ genotypes was 58.2% (32/55), while 3.6% of CIN2+ lesions were negative to HPV (Table 3).

A total of 94.6% of patients (52/55 cases) with histologically confirmed CIN2+ had positive immunostaining for p16/Ki67. A total of 15.1% of patients (21,139) with histologically proven CIN1 and 11.5% of patients (15/130) with negative histology had positive staining for p16/Ki67 (χ^2^ = 153.913; *p* < 0.001) OR: 114.34 (95% CI 33.93–385.37) (Table 4).

The overall sensitivity of p16/Ki67 immunostaining for the detection of CIN2+ was 94.5% (95% CI: 91.3–96.7), the specificity (%) was 86.6 (95% CI: 82.3–90.0), the PPV (%) was 59% (95% CI: 53.5–64.5) and the NPV (%) was 98.7 (95% CI: 96.6–99.6) (Table 5).

The HPV test showed a sensitivity (%) of 96.4 (95% CI: 93.5–98.0%) for CIN2+, a specificity (%) of 62.8 (95% CI: 57.3–68.1), a PPV (%) of 35 (95% CI: 29.5–40.1) and a NPV (%) of 98.9 (95% CI: 96.7–99.6).

## 4. Discussion

The diagnosis of CIN2+ in post-menopausal age is also made more difficult by the decline in the role of colposcopy in elderly women due to the low disease rate, the predominance of non-HPV16 genotypes and the lack of visualization of SCJ [15]. The use of the hrHPV test as a primary screening tool with high sensitivity and low specificity for the prevention of cervical cancer in women over 30 years old, and of the cytology as a triage strategy for positive cases proved to be very effective in women over 30 years of age but not in post-menopausal women [16]. Elderly women have some characteristics that make triage with cytology ineffective and modify the performance of the HPV test: the prevalence of HPV infections decreases with age; thus, in post-menopausal women, the HPV test will have a greater sensitivity and specificity similar to that of cytology but the atrophy of the squamous epithelium makes the Pap test less efficient and has the disadvantage of having a low specificity [17,18]. Some authors have reported a sensitivity of cytology for CIN2+ of 67% but this sensitivity drops to 17% for cases with atrophy [19].

Other authors have demonstrated that cytology is not an appropriate method for the diagnosis of CIN2+ in this age group, confirming the poor correlation between cytology and histology [20]. In Hermansson’s study, cytology identified only 3 CIN2+ out of 22 histologically confirmed CIN2+ [20].

Most CIN2+ histological lesions in post-menopausal women are not detected using the Pap smear test.

An additional marker is needed as a triage for post-menopausal ASC-US women such as p16/Ki67 double-stained cytology and HPV16 genotyping.

In the literature, numerous authors have studied the double staining cytology, and reported that it has a sensitivity comparable to the HPV test but with a higher specificity [21,22,23]. This technique has been little studied in women with post-menopausal ASC-US cytology. In contrast to p16 immunohistochemistry, the double staining technique is not based on cell morphology that, on the one hand, eliminates the reader-dependent variability for which it offers more objective and therefore more reproducible data for the triage of women with ASC-US, while on the other hand, it is possible to obtain high sensitivity levels since only one cell dual-stained positive is sufficient for a positive test result.

In our study population, 55 cases (16.9%) showed CIN2+ histological lesions. The high detection rate of CIN2+ can be attributed to several factors. One reason is that our study population comes from a second-tier center where only positive cases are referred. Secondly, the sample under study consists of postmenopausal women in whom cytology loses its effectiveness for diagnosis. There was a significant trend (*p* < 0.001) for p16/Ki67 positivity and increasing histological severity of the lesion: this was observed in 11.5% of histology-negative cases, in 18.7% of CIN1 cases, and in 94.6% of CIN2+ cases detected on histology.

Our study found that the specificity of p16/Ki67 immunocytochemistry for CIN2+ detection was higher than the HPV DNA test (χ^2^ = 153.913 *p* < 0.001). In particular the use of p16/Ki67 provides a 94.5% sensitivity level (95% CI 91.3–96.7) to detect CIN2+, very close to the 96.4% sensitivity of the HPV test (95% CI 93.5–98) but with greater specificity (86.6%) (95% CI 82.3–90.0), leading to a higher PPV (59%) for CIN2+.

In summary, the HPV test may be superior to stratify the long-term risk of cervical neoplasms, while the p16/Ki67 test may be particularly strong in predicting an immediate outcome; moreover, the p16/Ki67 test could allow longer intervals in HPV-positive women but who are negative at the triage test.

Our results on the use of P16/Ki67 in post-menopausal ASC-US women are comparable to the data of other authors who have studied it in women of childbearing age, leading to the conclusion that, as in childbearing age and in post-menopause, p16/Ki67 offers significantly high sensitivity and specificity in the triage of ASC-US women for CIN2+ [21,22,23].

The clinical efficacy of the HPV test, which only detects the presence of the virus in the cell, is variable and depends on the age of the woman [15,24]. In fact, the high prevalence of HPV infections in younger age groups makes the use of hrHPV testing limited with low specificity. In post-menopause, the low prevalence of HPV infection increases the specificity of the HPV test, while the cytology in this cohort has the disadvantage of having a low specificity causing an increase in ambiguous diagnoses, such as ASC-US. This indicates that while HPV testing and cytology depend on the woman’s age, dual staining is highly specific for cell cycle dysregulation.

Another way to increase the specificity of the HPV test comes from the further stratification of ASC-US-positive women based on HPV16 genotyping [25,26].

Since HPV16 and HPV 18 are associated, respectively, with 53.5% and 17.2% of all invasive cervical carcinomas, HPV16–18 genotyping could identify women at higher risk of CIN2+ and provide a clinically useful stratification of disease risk [27,28]. In our series of post-menopausal women, we only used HPV16 genotyping because we did not have any CIN2+ lesions HPV 18 positive, and we had only two cases of HPV 18 but in low-grade lesions.

In our previous study conducted on ASC-US women of childbearing age, the prevalence of HPV16 was 65.6% with 81.9% of CIN2+ cases; in our current study sample of post-menopausal women, the prevalence of HPV16 was 48.4% with a prevalence in CIN2+ women of 28.4% (21/74) [29].

In our study of post-menopausal women, 39.6% (21/53) of CIN2+ lesions were HPV16 positive, while 60.4% (32/53) had hrHPV genotypes; the proportion of cervical lesions that were HPV16 positive seems to decrease substantially with age (the two cases of invasive cancer were not HPV 16 positive) [15]. Another author, Wright et al. states that for women of childbearing age with HPV 16 who have had a normal Pap smear, the likelihood of developing precancerous cervical lesions is 35 times higher compared to women with other genotypes [30].

The presence of the HPV16 genotype in our previous study (women of childbearing age) was associated with a five times greater risk of developing a high-grade lesion (CIN2+) OR = 4.62 (95% CI: 3.13–6.82); the current study (post-menopausal women) makes us deduce that HPV16 genotyping with an OR = 0.60 (95% CI 0.30–1.18), *p* = 0.139 does not allow us to discriminate between CIN2+ women and CIN2- (negative, CIN1) women; therefore, the genotyping may not be the best strategy in older women [25].

Based on our data, we can conclude that given the low sensitivity of cytology and the low percentage of HPV16-positive cancers among elderly women, triage by cytology and genotyping is not the best strategy in elderly women; the double staining cytology shows high profiles of sensitivity and specificity for CIN2+ in ASC-US triage both in young and post-menopausal women.

The strength of this study is that it is the first retrospective analysis to study the utility of dual staining in ASCUS triage in postmenopausal women.

We also considered a sample of post-menopausal women for at least two years with restrictive inclusion criteria, with cervical and conical biopsy samples instead of curettage samples; this allowed the evaluation of both cytology and the architectural relationship with the basement membrane.

We investigated the genotype and correlation between HPV16 and CIN2+.

The limitations of our research are mainly related to the small sample size and its retrospective nature. Another potential bias could be that the study population selected was from a second-tier center; this could lead to slightly different results than a general population.

Further prospective studies with multiple post-menopausal women are needed to confirm the current findings.

## Figures and Tables

**Table 1:** The ASC-US cases related to the results of the histological examination and HPV status.

	Negative	CIN1	CIN2	CIN3	Ca	Overall
HPV positive	19 (42.3%)	81 (58.3%)	34 (97.1%)	17 (94.4%)	2 (100%)	153 (47.3%)
HPV negative	111 (57.7%)	58 (41.7%)	1 (2.9%)	1 (5.6%)	0 (0%)	171 (52.7%)
Total	130	139	35	18	2	324

CIN, Cervical Intraepithelial Neoplasia; Ca, Carcinoma. hrHPV: all hrHPV genotypes.

**Table 2:** Stratifying the sample HPV positive under examination according to genotyping.

Genotype	Negative	CIN1	CIN2	CIN3	Ca	Overall
HPV16	5 (26.3%)	48 (59.3%)	14 (41.2%)	7 (41.2%)	0 (0%)	74 (48.4%)
hrHPV	14 (73.7%)	33 (40.7%)	20 (58.8%)	10 (58.80%)	2 (100%)	79 (51.6%)
Total	19	81	34	17	2	153

CIN, Cervical Intraepithelial Neoplasia; Ca, Carcinoma. hrHPV: all hrHPV except HPV16.

**Table 3:** Prevalence of HPV status in postmenopauasal women with CIN2+ histological lesions.

HPV Status	CIN2	CIN3	Ca	CIN2+
HPV 16	14 (40%)	7 (38.9%)	0%	21 (38.2%)
hrHPV	20 (57.1%)	10 (55.5%)	2 (100%)	32 (58.2%)
negative	1 (2.9%)	1 (5.6%)	0%	2 (3.6%)
Total	35	18	2	55

CIN, Cervical Intraepithelial Neoplasia; Ca, Carcinoma. hrHPV: all hrHPV except HPV16.

**Table 4:** Study group divided according to histological results (Negative, CIN1, CIN2, CIN3) and the tests used (p16/Ki67, HPV DNA).

Test	Negative	CIN1	CIN2	CIN3	Ca	Overall
P16/Ki67						
positive	15	21	32	18	2	88 (27.2%)
negative	115	118	3	0	0	236 (72.8%)
HPV test DNA						
positive	19	81	34	17	2	153 (47.3%)
negative	111	58	1	1	0	171 (52.7%)
Overall	130 (40.1%)	139 (42.9%)	35 (10.8%)	18 (5.5%)	2 (0.6%)	324

CIN, Cervical Intraepithelial Neoplasia; Ca, Carcinoma. hrHPV: all hrHPV except HPV16.

**Table 5:** Sensitivity, Specificity, PPV and NPV of the HPV test and p16/Ki67 and OR for CIN2+ in postmenopausal women with ASCUS cytology.

Test	Histology	Sensitivity	95% CI	Specificity	95% CI	PPV	95% CI	NPV	95% CI	OR
p16/Ki67	CIN2+	94.5	91.3–97	86.6	82.3–90.0	59	53.5–64.5	98.7	96.6–99.6	114.34
HPVDNA	CIN2+	96.4	93.5–98	62.8	57.3–68.1	35	29.5–40.1	98.9	96.7–99.6	44.79

PPV, Positive Predictive Value; NPV, Negative Predictive Value; OR, Odds R.

## Data Availability

There are no linked research data sets for this paper. Data will be made available on request.

## Supplementary Materials

The following supporting information can be downloaded at: https://www.mdpi.com/article/10.3390/jcm12103400/s1, Figure S1: HPV16 genotyping in postmenopausal women cannot be used to increase HPV test specificity, double staining is an important aid in the management of postmenopausal ASCUS patients, offering a sensibility equal to the HPV Test but with greater specificity.
