# Supplementary material for: Performance of p16/Ki67 Immunostaining for Triage of Elderly Women with Atypical Squamous Cells of Undetermined Significance
Source: J Clin Med. 2023 May 11;12(10):3400. doi: 10.3390/jcm12103400 (PMC10218909; doi:10.3390/jcm12103400)
Supplement: Supplementary file 1 [file jcm-12-03400-s001.zip › jcm-2288009-supplementary.pdf]

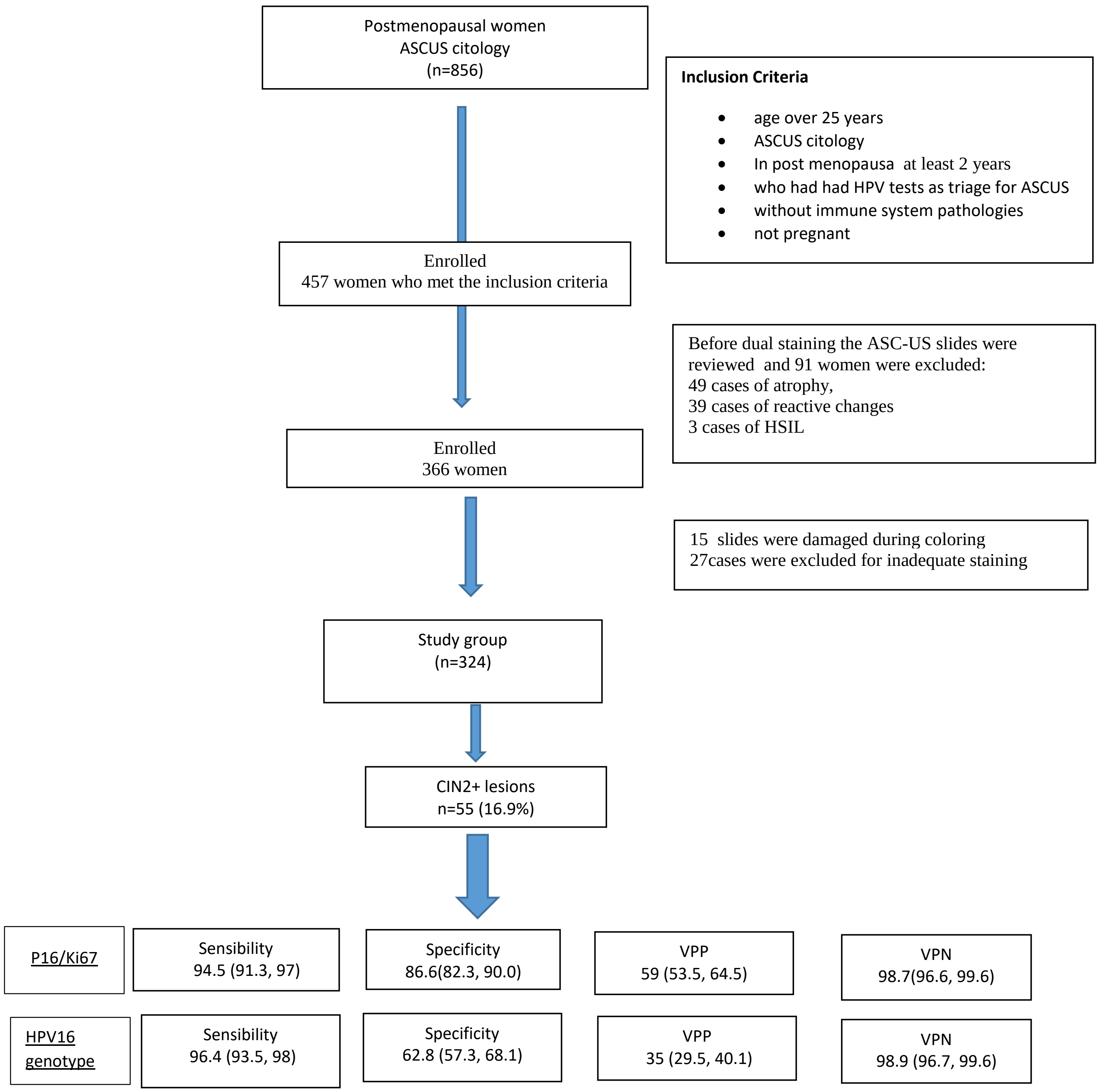

Figure S1. HPV16 genotyping in postmenopausal women cannot be used to increase HPV test specificity, double staining is an important aid in the management of postmenopausal ASCUS patients, offering a sensibility equal to the HPV Test but with greater specificity.
